# Immune Enhancement of Clam Peptides on Immunosuppressed Mice Induced by Hydrocortisone

**DOI:** 10.3390/molecules28155709

**Published:** 2023-07-28

**Authors:** Meibin Lv, Mengyue Liu, Shengcan Zou, Dongli Yin, Chenghan Lv, Fei Li, Yuxi Wei

**Affiliations:** 1 College of Life Sciences, Qingdao University, Qingdao 266071, China; lmb19846950833@163.com (M.L.); lmy02112021@163.com (M.L.); 2 Qingdao Chenlan Pharmaceutical Co., Ltd., Qingdao 266105, China; sczou@chenland.com (S.Z.); dongli215@163.com (D.Y.); chenghanlyu@126.com (C.L.)

**Keywords:** clam peptides, immunomodulatory, gut microbiota, immunosuppressed mice, hydrocortisone

## Abstract

Clam peptides, marine-derived biological peptides, have been broadly investigated and applied as health foods, among which immunomodulation is one of their biological activities that cannot be ignored in vivo. In this study, we concentrated on exploring the effects of *Ruditapes philippinarum* peptides (RPPs) on immunomodulation and the balance of intestinal microbiota in hydrocortisone (HC)-induced immunosuppressed mice. The results revealed that RPPs could increase the thymus and spleen indices and number of white blood cells, promote the secretion level of cytokines (IL-2, IL-6, TNF-α, and INF-γ), repair the morphology of the spleen and thymus, and enhance the proliferation of T-lymphocyte subsets in immunosuppressed mice. Moreover, RPPs improved the abundance of beneficial bacteria and preserved the ecological equilibrium of the gut microbiota. In conclusion, RPPs have significant immunomodulatory effects on immunosuppressed mice and may be developed as immunomodulators or immune adjuvants in functional foods and drugs; they are also beneficial to the utilization of the high value of marine shellfish.

## 1. Introduction

Immunity is the organism’s protective response to recognize and counteract foreign invaders such as pathogens and eliminate detrimental stimuli and tumor cells [1]. Currently, many people’s immune function is gradually weakened due to their sub-healthy lifestyle, accompanied by the occurrence of various immune disorders, such as malignant tumors [2], chronic kidney disease [3], rheumatism [4], obesity [5], and diabetes [6]. Glucocorticoids (GCs) have a unique niche in the treatment of inflammatory and autoimmune diseases on account of their obvious immune-suppressive, anti-shock, and anti-inflammatory bioactivities [7]. Hydrocortisone, a kind of glucocorticoid for the treatment of inflammatory diseases, may cause serious adverse effects (immunosuppression, hypertension, and osteoporosis) during long-term and excessive use [8,9,10]. Therefore, in this study, a large dosage of hydrocortisone was used to establish an immunosuppressive model to investigate the effect of natural active peptides on immunosuppression.

Modern pharmacological studies have certified that peptides, also called active peptides, possess diverse functionalities, such as immune enhancement [11], hypotension [12], antioxidant effects [13], and the regulation of intestinal flora [14]. It has been documented that low-molecular-weight protein hydrolysates and peptides containing hydrophobic amino acids can promote immune regulation [15]. Our laboratory has performed analysis on the peptide sequence composition of RPPs, and these sequences primarily include IIVDELK, VFPSRR, ALFLK, ILFIR, VVTAVK, and EIAQDFK (unpublish data). In particular, one of the most vital bioactivities of food-derived bioactive peptides is the immunomodulatory activity, which has already been extensively reported. For example, peptides derived from Chinese medicine can enhance intestinal immunity and adjust the balance of cecum intestinal flora in immunosuppressed mice [16]. Pan et al. concluded that hydrolysates from milk protein could boost immune function in mice by reducing the level of IL-4 and increasing growth factor-β levels in serum [17]. Hydrolysates obtained from fish eggs proteins could strengthen the splenic NK cell cytotoxicity and content of IgA in the gut and promote the proliferation of spleen lymphocytes [18].

Clams, an important marine resource, belong to the gene *Ruditapes* and the family *Veneridae*, which are broadly distributed in the coastal areas of China, Korea, Japan, and Spain. Notably, *Ruditapes philippinarum,* a treasure of the sea for the prevention of human diseases (chronic bronchitis, accumulation of sputum, dry cough, etc.), has a long history in oriental folk medicine. In China, the germplasm and culture resources of *Ruditapes philippinarum* are abundant, but their bioactivities have not been fully exploited and utilized because the published literature regarding the medicinal value of *Ruditapes philippinarum* is still relatively limited compared to the examinations of breeding techniques, genetic breeding, morphological structure, pathologic toxicology, etc. [19]. Modern pharmacological studies have revealed that *Ruditapes philippinarum* peptides (RPPs), a major bioactive component in the *Ruditapes philippinarum*, had diverse bioactivities, such as anticoagulant, antihypertensive, and anticancer, etc. [20]. Nevertheless, it is unclear whether RPPs improve immunosuppression effects in vivo. Herein, the aim of this study was to evaluate the immune enhancement capacities of RPPs in mice that had been immunosuppressed by HC. The present study outlined that RPPs possessed experimental evidence of immune enhancement activity and verified that RPPs have enormous potential to become natural immunity modulators in the realms of functional foods.

## 2. Results

### 2.1. Effects of RPPs on Thymus and Spleen Indices in Immunosuppressed Mice

**Figure 1:**
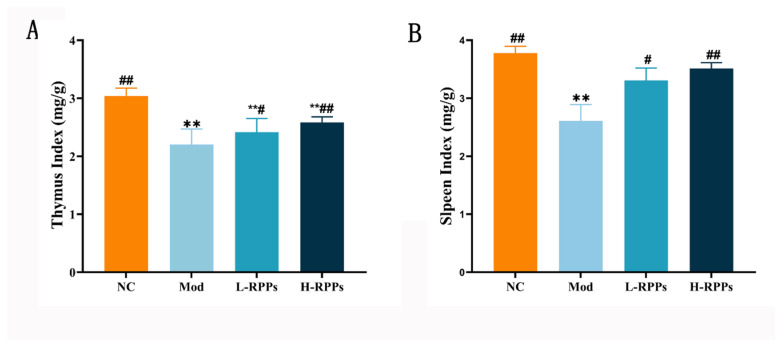
Effect of RPPs on immune organ indices in immunosuppressed mice. (**A**) Thymus indices; (**B**) Spleen indices. ## *p* < 0.01 and # *p* < 0.05 compared with the Mod group; ** *p* < 0.01 compared with the NC group.

The spleen and thymus are the main immune system organs. Additionally, the indices of immune organs can reflect the immune function of BALB/c mice induced with HC to some extent. According to Figure 1A,B, the Mod group showed a significant decrease in thymus and spleen indices (*p* < 0.01), demonstrating the successful establishment of the immunosuppressed model. In addition, the L-RPPs and H-RPPs groups exhibited significantly higher spleen and thymus indices than the Mod group (*p* < 0.01; *p* < 0.05), and this effect was found to be dose-dependent. These results suggest that RPPs are capable of effectively reversing immune organ atrophy induced by HC.

### 2.2. Effect of RPPs on Blood Indices in Immunosuppressed Mice

**Figure 2:**
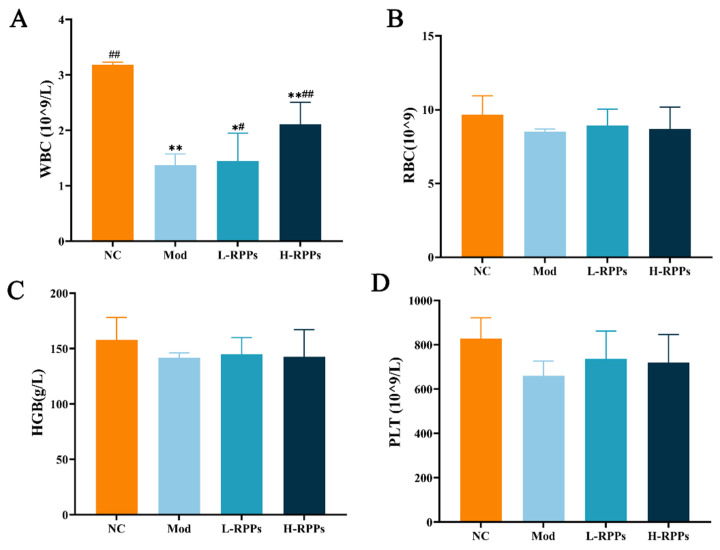
Effect of RPPs on complete blood cell count in immunosuppressed mice. (**A**) White blood cell count (WBC); (**B**) red blood cell count (RBC); (**C**) platelet count (PLT); (**D**) hemoglobin content (HGB). ## *p* < 0.01 and # *p* < 0.05 compared with the Mod group; ** *p* < 0.01 and * *p* < 0.05 compared with the NC group.

RBCs are a component of the immune system, while WBCs are essential immune cells. The number of WBCs was significantly reduced in the Mod group, as depicted in Figure 2A. However, when L-RPPs and H-RPPs were administered, the number of WBCs significantly increased (*p* < 0.05 or *p* < 0.01) and displayed a dose-dependent effect. Although the Mod group exhibited a decrease in HGB, PLT, and RBCs, there was no statistically significant difference observed compared to both the control group and the RPPs group (Figure 2B–D). These findings indicated that RPPs have the potential to restore the number of immune cells.

### 2.3. Effect of RPPs on the Levels of Cytokines in Immunosuppressed Mice

**Figure 3:**
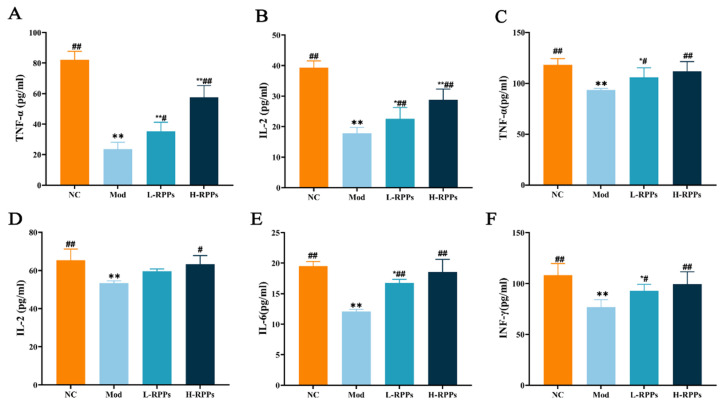
Effect of RPPs on cytokine levels in immunosuppressed mice. Effect of RPPs on TNF-α and IL-2 in serum (**A**,**B**) and TNF-α, IL-2, IL-6 and INF-γ in spleen (**C**–**F**). ## *p* < 0.01 and # *p* < 0.05 compared with the Mod group; ** *p* < 0.01 and * *p* < 0.05 compared with the NC group.

As exhibited in Figure 3A,B, the contents of IL-2 and TNF-α in the serum were significantly lower in the Mod group (*p* < 0.05). Compared to the Mod group, both the L-RPPs and H-RPPs groups indicated an obvious increase in the concentrations of IL-2 and TNF-α (*p* < 0.05 or *p* < 0.01). Moreover, the serum cytokine levels exhibited a dose-dependent increase with RPPs treatment. Figure 3C–F indicate that the Mod group had considerably reduced levels of the spleen cytokines (INF-γ, IL-6, TNF-α, and IL-2). However, compared to the Mod group, the H-RPPs group showed significantly higher levels of TNF-α, IL-6, IL-2, and INF-γ (*p* < 0.05 or *p* < 0.01). The L-RPPs group showed significant increases in TNF-α, IL-6, and INF-γ levels compared to the Mod group (*p* < 0.05), whereas the IL-2 levels showed a slight increase but with no significant difference. These findings suggested that RPPs could enhance the immune response in immunodeficient mice by increasing the levels of inflammatory factors in the serum and spleen homogenates.

### 2.4. Effect of RPPs on Histopathological Changes in Immune Organs in Immunosuppressed Mice

**Figure 4:**
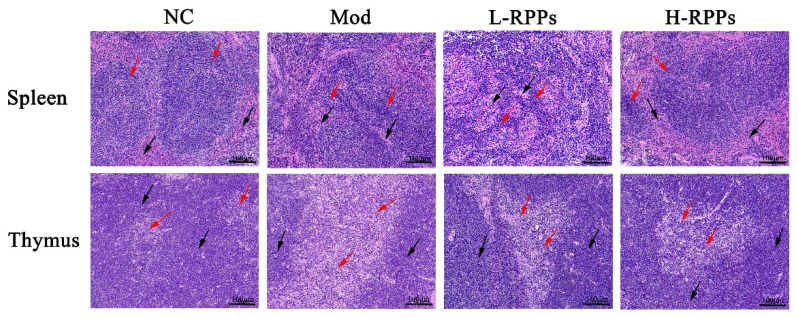
Effect of RPPs on the histological morphology of spleen and thymus in immunosuppressed mice. Hematoxylin and eosin staining. Bars = 100 μm, magnification, 20×. In spleen sections, the red arrows represent white pulp, while the black arrows represent red pulp. In thymus sections, the red arrows represent the medulla, while the black arrows represent the cortex.

In Figure 4, the thymus and spleen’s histological morphology was depicted. The spleen of the mice in the NC group displayed an intact structure with closely spaced splenocytes and distinct separating borders between the white and red pulp, according to the histological findings. Inversely, there were no significant dividing boundaries between the white and red pulp of the spleen in the Mod group, and the splenocytes were irregularly arranged. Compared with the Mod group, the detection of the spleen histological morphology did not improve significantly by H&E staining in the L-RPPs group. Instead, in the H-RPPs group, the spleen structure appeared to be relatively intact, characterized by a clear boundary between the red and white pulps and an increased number of lymphocytes arranged regularly.

The Mod group exhibited thymus gland atrophy, cortical thinning, and the expansion of the medullary substance, as revealed by H&E staining. In comparison with the Mod group, the RPPs groups could restore the thymus damage to a certain degree. For example, the thymic lobules tended to be normal, the cortical part was significantly increased, and the medullary part was significantly reduced. These results demonstrated that RPPs improved the spleen and thymus tissue damage caused by HC.

### 2.5. Effect of RPPs on T Cell Expression in Immunosuppressed Mice

**Figure 5:**
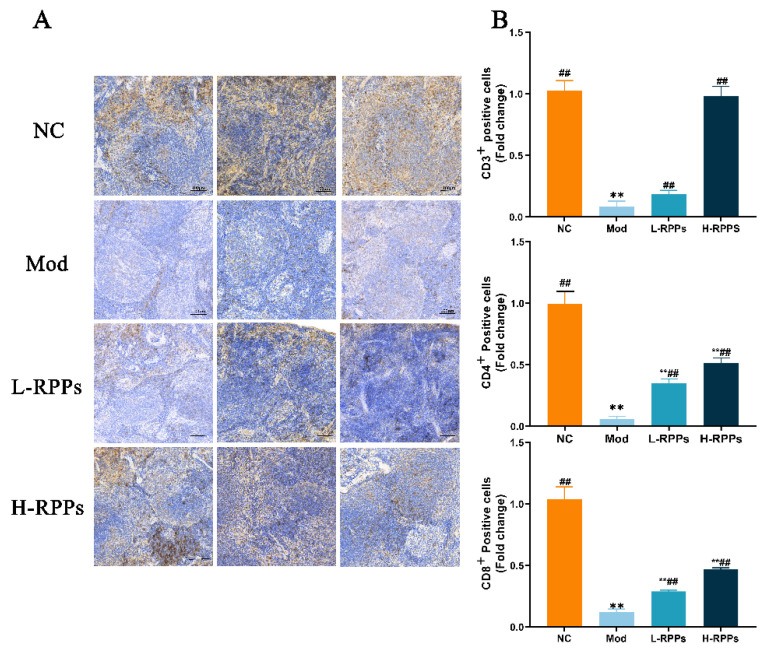
Effect of RPPs on CD3^+^, CD4^+^, and CD8^+^ T cell expression (**A**) and IOD values (**B**) in the thymus and spleen in immunosuppressed mice (Bars = 100 μm, magnification, 20×). ## *p* < 0.01 compared with Mod group; ** *p* < 0.01 compared with NC group.

Immunohistochemistry data were used to demonstrate that the expression levels of the surface markers CD3^+^, CD4^+^, and CD8^+^ on spleen cells in the Mod group were significantly reduced. In contrast, the RPPs group displayed significantly higher expression levels of these markers (Figure 5A). The integral optical density (IOD) semi-quantitative results of immunohistochemistry experiments are shown in Figure 5B. The Mod group showed significantly lower levels of CD3^+^, CD4^+^, and CD8^+^ expression on splenocyte surfaces (*p* < 0.01). The Mod group showed a significant decrease in the expression levels of CD3^+^, CD4^+^, and CD8^+^ on the surfaces of splenocytes (*p* < 0.01). However, after RPPs administration, the expression levels of CD3^+^, CD4^+^, and CD8^+^ on the surfaces of splenocytes were significantly elevated in comparison to the Mod group (*p* < 0.01). The findings suggested that the immune-enhancing effect of RPPs on immunosuppressed mice might be related to cellular immunity.

### 2.6. Effect of RPPs on the Gut Microbiota Composition in Immunosuppressed Mice

**Figure 6:**
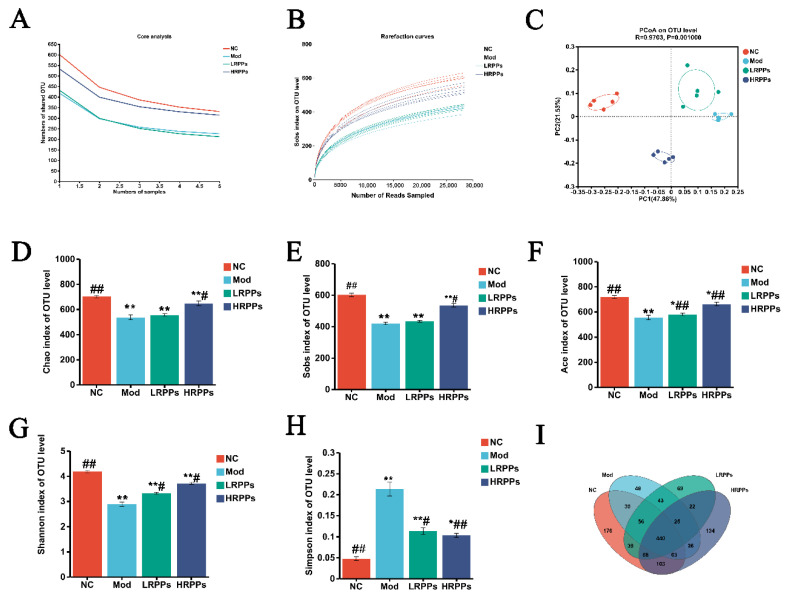
Effect of RPPs on structure of gut microbiota in immunosuppressed mice. (**A**) Core curve; (**B**) Dilution curve; (**C**) PCoA of the intestinal flora based on Bray-Curtis distance; (**D**–**H**) Alpha diversity presented by Shannon, Simpson, Sobs, Ace, and Chao1 indices; (**I**) Venn map. Different colors represent different groups. ## *p* < 0.01 and # *p* < 0.05 compared with Mod group; ** *p* < 0.01 and * *p* < 0.05.

The influence of RPPs’ on the structure of the intestinal microbiota in immunosuppressed mice was examined using high-throughput sequencing of 16S rRNA. The core curve flattened over a number of samples, demonstrating that enough samples were sequenced, and the same result was observed in the dilution curve (Figure 6A,B).

The alpha-diversity analysis mainly included Sobs, Ace, Chao1, Shannon, and Simpson indices. Among them, Ace, Chao1, and Sobs indices show the percentage of bacterial abundance, while the Shannon and Simpson indices primarily reflect the variety of intestinal flora [21]. As indicated in Figure 6D–G, the Sobs, Ace, Chao1, and Shannon indices were dramatically reduced in the Mod group (*p* < 0.05). The bacterial abundance and alpha diversity analyzed with the Sobs, Ace, Chao1, and Shannon indices were greatly raised in the RPPs treatment group (*p* < 0.05). As displayed in Figure 6H, the Simpson index exhibited higher diversity in the Mod group (*p* < 0.05). Notably, RPPs treatment can repair bacterial abundance and diversity. The Simpson indices were significantly reduced in the RPPs-treated group (*p* < 0.05), indicating that RPPs could ameliorate the reduction in intestinal diversity in HC-induced immunosuppressed mice.

**Figure 7:**
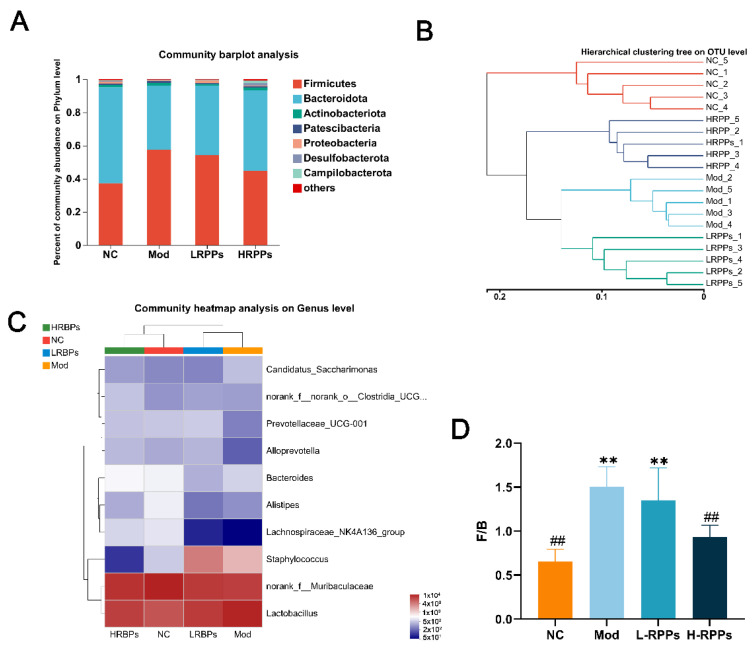
RPPs promote the balance of gut microbiota in immunosuppressed mice. (**A**) Taxonomic analysis of bacteria at the phylum level; (**B**) hierarchical cluster analysis diagram. (**C**) taxonomic analysis of bacteria at the genus level; (**D**) ratio of F/B in the gut. ## *p* < 0.01 compared with Mod group; ** *p* < 0.01 and compared with NC.

Sequences are clustered according to a 97% similarity, and each class formed is called an OTU. As exhibited in Figure 6I, there were 440 OTUs that coexisted among the NC, Mod, L-RPPs, and H-RPPs groups. A total of 589, 601, and 672 OUTs were shared between the NC and Mod groups as well as the L-RPPs and H-RPPs groups, respectively. Additionally, both the Bray–Curtis intersample distance matrix and OUT abundance data were applied to principal coordinate analysis (PCoA), exhibiting the gut microbial community for each group. The gut microbiota of the NC group differed in species composition and clusters far from the Mod group (Figure 6C), while the H-RPPs treated group and NC groups were more similar in the structure and clusters of the intestinal microbiota. Furthermore, beta diversity analysis revealed that the H-RPPs group’s hierarchical clustering tree on the OUT level differed greatly from the Mod group and was much more similar to the NC group (Figure 7B). The findings demonstrated that RPPs intervention might make the OUTs between the RPPs group and the NC group more similar.

To investigate the specific alterations in intestinal microbial species, the taxonomic composition of the microbiota was examined at the phylum and genus levels; the results are presented in Figure 7A. The dominant phylum in the microbiota was *Firmicutes*, *Bacteroides*, *Aspergillus*, and *Actinobacteria*, among which *Firmicutes* and *Bacteroidetes* went represented over 90% of them. In comparison to the NC group, the Mod group exhibited an increased relative abundance of *Firmicutes* from 37.33% to 57.48%, and the abundance of *Bacteroidetes* decreased from 57.85% to 38.66% in the Mod group. Compared to the Mod group, the relative abundance of *Firmicutes* decreased from 57.48% to 44.88%, and the abundance of *Bacteroidetes* increased from 38.66% to 48.53% in the H-RPPs groups. The *Firmicutes* to *Bacteroidetes* (F/B) ratio was significantly higher in the Mod group, whereas RPPs partially reversed this change (Figure 7D). We also investigated the relative abundance of intestinal microbiota at the genus level (Figure 7C): *norank_f_Murbaculaceae*, *Lactobacillus*, *Staphylococcus*, *Bacteroides*, *Prevotellaceae_UGG-001*, and *Alistipes*, *Alloprevotalla* were the dominant gut microbiota. In addition, *norank_f_Murbaculaceae*, *Lachnospiracea_NK4A136_group*, and *Prevotellaceae_UGG-001* decreased; *Staphylococcus* and *Candidatus_Saccharimonas* were increased in the Mod group. However, RPPs treatment may mitigate the negative effects of HC in mice; the abundance of *norank_f_Murbaculaceae*, *Lachnospiracea_NK4A136_group*, and *Prevotellaceae_UGG-001* was recovered, and *Staphylococcus* and *Candidatus_Saccharimonas* were reduced. These results manifested that RPPs treatment could improve the unbalanced flora in the gut and increase the diversity and abundance of sample species in HC-induced immunosuppressed mice.

## 3. Discussion

Recently, bioactive peptides have been paid more and more attention due to their various bioactivities. Notably, the search for peptides with good immune regulation among the natural bioactive peptides is one of the hot research topics. Therefore, we focused on RPPs from *Ruditapes philippinarum*, studying its immunomodulatory effect on HC-induced immunosuppressed mice.

Cyclophosphamide has been widely used to establish immunosuppressed mice models, but our previous study found that RPPs have strong ACE inhibitory activity, and combined treatment with cyclophosphamide could cause mouse death (unpublished data). For this reason, our present study has committed to finding new immunosuppressants with non-toxic side effects. Glucocorticoids (e.g., HC) can be used to build immunosuppressed models by inhibiting various stages of the immune process. Therefore, in our study, HC was selected to induce immunosuppression in mice [22,23]. Our findings revealed that HC might lower the immune organ index, inhibit the proliferation of leukocytes, decrease the expression levels of cytokines, damage the histological morphology of immune organs, and disrupt the balance of the intestinal microbiota, which were consistent with immunosuppressed mice induced by cyclophosphamide [24,25,26].

It is widely acknowledged that immune organs, immune cells, and immunological substances make up the immune system [24]. The main immunological organs are the spleen and thymus, and both organs’ indices and morphologies can indicate how well they perform innate immunity [23,27]. Previous research has indicated that hydrolyzed polysaccharides from mussels may enhance immune function and increase immune organ indices in immunosuppressed mice [28]. In the present study, RPPs can enhance the immune organ indices, and their pathological morphologies were improved after RPPs treatment. The results indicate that RPPs can enhance the immune system by promoting the strengthening of immune organs. Tang et al. also discovered that polysaccharides derived from *Lignosus rhinocerotis sclerotia* had the potential to enhance the immune response in the host, and they observed a significant improvement in the spleen index [29]. These outcomes matched the conclusions reached by Khan et al. They discovered that in immunosuppressed mice, shrimp peptide hydrolysate might repair damaged immunological organs, and it increased immune organ indices [30].

Furtherly, in this study, the immune-enhancement mechanisms of RPPs in immunosuppressed mice were investigated. The immune system’s primary regulatory cells are mature T lymphocytes. The markers for various T lymphocyte subsets include CD3^+^, CD4^+^, and CD8^+^ [31,32]. The CD4^+^ T cell is the representative of helper T cells, and the CD8^+^ T cell is the representative of cytotoxic T lymphocytes. CD4^+^ plays a significant role in autoimmune and inflammatory diseases because it assists B lymphocytes, macrophages, and T lymphocytes to activate their immune functions, and CD8^+^ specifically kills target cells [33,34]. The number of T lymphocyte subtypes remains stable when the body is operating normally, and an alteration of the number of T lymphocytes may result in immune dysfunction. Our results demonstrated that RPPs increased the number of leukocytes in mice treated with HC, suggesting a protective effect of RPPs against immunosuppressed. Furthermore, RPPs administration resulted in an increase in CD3^+^, CD4^+^, and CD8^+^ T cell populations, demonstrating the immunomodulatory effects of RPPs.

As vital immune response mediators and regulators, cytokines can reflect the body’s immune regulatory function by varying their secretion levels [35,36,37]. The proper operation of the body’s immune system can be controlled by the balance of Th1/Th2 homeostasis [38]. IL-2 regulates immune function by encouraging B lymphocyte proliferation and differentiation and inducing lymphokine activation in killer cells [39,40,41]. The immune response of T and B cells can be controlled by certain immune factors, such as IL-6 [42]. INF-γ enhances antibacterial immunity by acting on macrophages and T cell development [43]. TNF-α is a cytokine secreted by macrophages, which is mainly involved in immune response and the expression of inflammatory mediators [44]. The immune regulatory effects are likely mediated through the activation of complex immune regulatory mechanisms involving macrophages and lymphocytes [2]. Peptides can activate signaling cascades within immune cells through various receptors, such as Toll-like receptor 4 (TLR-4). The activation of TLRs can promote the expression of cytokines like IL-2 and TNF-α, thereby enhancing both cellular and humoral immune responses [45]. Zhao et al. found that low-molecular-weight peptides from red shrimp (*Solenocera crassicornis*) can improve the body’s immune response by increasing the secretion of TNF-α and TFN-γ [46]. The findings of this investigation indicate that RPPs can strengthen the expression of IL-2 and TNF-α in serum and the levels of IL-2, IL-6, INF-γ, and TNF-α in spleen homogenate in HC-treated mice, indicating that RPPs can enhance the immunity of mice by regulating the secretion of cytokines. Similar results have been documented in other studies, where the ingestion of peptides resulted in a significant elevation of IL-2 and INF-γ levels in immunocompromised mice [47].

The intestinal microbiota contains a wide variety of bacteria, and the host immune function is influenced by the composition and metabolites of these bacteria. Furthermore, disruption of immune homeostasis can lead to dysbiosis of the microbiota [48]. The structure of gut microbiota can reflect the health of the host to a certain extent. In fact, extensive research has demonstrated that bioactive peptides play vital roles in regulating gut microbiota, which in turn regulate host immunity [26,47,49]. For example, Cui et al. confirmed the regulatory impact of peptides from traditional Chinese medicine by enzymatic hydrolysis on gut microbiota [50]. This investigation found that RPPs could affect the gut microbiota in HC-treated mice by using 16S rRNA sequencing. In detail, Shannon, Simpson, Ace, and Chao1 indices were utilized to evaluate the microbial composition diversity, and these indices were significantly elevated after RPPs administration. Through the Venn map, we could find the OUT similarity between the RPPs group and the NC group. These results indicated that RPPs can enhance the diversity and abundance of gut microbiota in immunosuppression mice. Principal coordinate analysis (PCoA) revealed significant alteration in the microbial community structure in the Mod group, and this negative effect was reduced in the RPPs group, among which the composition of the intestinal microbial structure in the H-RPPs group was closer to that in the NC group. Principal coordinate analysis (PCoA) revealed a significant alteration in the microbial community structure in the Mod group, and this negative effect was reduced in the RPPs group, among which the composition of intestinal microbial structure in the H-RPPs group was closer to that in the NC group. This is in agreement with Chen’s findings that polysaccharides from the flowers of tea (*Camellia sinensis* L.) can improve the structure of intestinal flora in immunosuppressed mice [51].

At the phylum and genus levels, the structure and composition of the bacteria in the gut were further explored. At the phylum level, *Bacteroidetes* and *Firmicutes* were the most common phylum among all taxa. In our study, it was found that RPPs can reduce gut flora imbalance and boost the population of helpful bacteria in immunosuppressed mice. This aligns with findings from earlier investigations that found that polysaccharides obtained from the roots of *Millwttia* could regulate the contents of *Bacteroidetes* and *Firmicutes* [52]. On a genus level, in contrast to the NC group, *norank_f_Muribaculaceae*, *Bacteroides*, *Prevotellaceae_UGG-001*, *Alistipes*, *Lachnospiracea_NK4A136_group*, *Alloprevotella*, and *Odoribacter* were reduced in the Mod group, but this change was significantly reversed by treatment with RPPs. *Bacteroides*, *norank_f_Muribaculaceae*, *Alloprevotella*, *unclassified_f_Lachnospiracea*, and *Lachnospiracea_NK4A136* have been reported to be producers of short-chain fatty acids (SCFAs) such as propionic, acetate, and butyric acids [39,53]. SCFAs control the development of intestinal epithelial cells and influence both innate and acquired immunity [54,55]. Moreover, the genus of *Alistipes* also has a protective effect on the organism during cancer and immunotherapy [56]. *Bacteroides* are one of the dominant genera of the microbiota in the intestines and maintain the stability of the gut microbial ecosystem [57]. The gut is the largest immune organ in the human body, and the integrity and permeability of the gut barrier are important indicators of gut health. Lipopolysaccharide (LPS) is produced and released into the bloodstream by the gut microbiota, increasing gut permeability and causing inflammation, which reflects the integrity of the gut mechanical barrier. The excessive release of LPS can disrupt the expression of intestinal tight junction proteins. Xiang et al. found that oyster (*Crassostrea gigas*) peptides can reduce the release of LPS and increase the expression of intestinal tight junction proteins, thereby improving the permeability and integrity of the gut barrier and regulating the composition of the gut microbiota [47]. Therefore, we speculate that RPPs may stimulate the intestinal immune system, thereby modulating systemic immunity. We speculate that RPPs may also improve the composition of the gut microbiota by repairing the intestinal barrier.

## 4. Materials and Methods

### 4.1. Materials

*Ruditapes philippinarum* was purchased from a neighborhood market (Qingdao, China). A certain amount of clam meat was taken as raw material and homogenized, and then pure water was added in a ratio of 1:2 (*w*:*w*) to form a liquid mixture. The pH was adjusted to 7.0, and then the liquid mixture underwent a complex protease at 58 °C for 6 h. Then, the mixture was immersed in a boiling water bath to inactivate the enzymes for 10 min. After cooling, it was centrifuged at 4000 r/min for 30 min. The supernatant was collected and then filtered separately through a microfiltration membrane [21]. The filtrate was freeze-dried to obtain RPPs. The molecular weight distribution of RPPs was measured by high-performance liquid chromatography (HPLC) with a purity of Mw (<1000 Da) >90% [58]. ELISA kits of interferon-γ (INF-γ), interleukin-2 (IL-2), interleukin-6 (IL-6), and tumor necrosis factor-α (TNF-α) were purchased from Jingmei Biotechnology Co., Ltd. (Nanjing, China). Hydrocortisone (HC) was purchased from Henan Runhong Pharmaceutical Co., Ltd. (Zhengzhou, China). Paraformaldehyde fixative was purchased from Saville Biotechnology Co., Ltd. (Changzhou, China).

### 4.2. Animals

Forty male BALB/c mice, aged five to six weeks, were obtained from SPF (Beijing) Biotechnology Co., Ltd. (Beijing, China; Certificate number: SCXK (Jing) 2019-0010). The Ethics Committee of the Medical College of Qingdao University approved all animal experimental procedures (Permit No. QDU-AEC-2023342), and they were conducted following the guidelines of the Care and Use of Laboratory Animals at Qingdao University.

### 4.3. Animal Experiment Design

**Figure 8:**
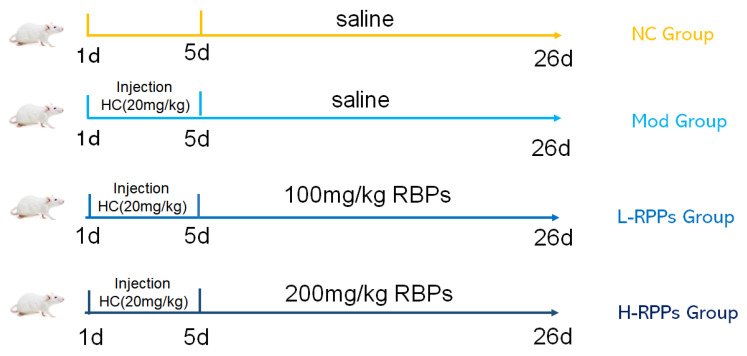
Protocol for the animal experiment.

Mice were randomly assigned to one of four groups (*n* = 10) after one week of adaptation to their new environment: the normal control (NC) group, the HC model (Mod) group, the HC + low-dose (L-RPPs, 100 mg/kg/day) group, and the HC + high-dose (H-RPPs, 200 mg/kg/day) group. A 20 mg/kg/day dose of hydrocortisone was given to each of the three groups, except for the NC groups, for five days in a row. From day 6 until day 21, the L-RPPs and H-RPPs groups were administered 100 and 200 mg/kg/day of RPPs, respectively, using a syringe with a gastric lavage needle to administer the prepared solution sample into the mouse’s stomach (as shown in Figure 8), and the NC and Mod groups received an equivalent volume of saline. The mice were weighed and fasted for 12 h with free access to water before being sacrificed by cervical displacement. Blood samples were collected from the eyeballs.

### 4.4. Calculation of Immune Organ Indices

After removal and weighing of the thymus and spleen, the immune organ index was calculated using the following formula:

Thymus or spleen index = weight of organ weight (mg)/body weight (g).

### 4.5. Complete Blood Cell Analysis

EDTA-coated tubes were used to collect the blood. Blood cell counts, including the white blood cell count (WBC), red blood cells (RBC), platelet count (PLT), and hemoglobin content (HGB), were measured using a hematology cell analyzer (BV-5000Vet, Shenzhen Mindray Animal Medical Technology Co., Ltd., Shenzhen, China).

### 4.6. Measurement of Cytokines Level in Serum and Spleen Homogenates

After the mice were sacrificed by cervical dislocation, blood samples were collected and centrifuged at 15 min (3000 r/min) to obtain serum. The spleen (0.1 g of spleen tissue and 900 μL PBS) was homogenized at 4 °C, and then the supernatant was collected by centrifuging. Serum and spleen concentrations of cytokines (IL-2, TNF-α, IL-6, and INF-γ) were performed using ELISA kits. All experimental procedures were conducted following the guidelines and instructions provided by the manufacturer.

### 4.7. Pathology and Immunohistochemical Analysis

The thymus and spleen were fixed using conventional methods in 4% paraformaldehyde for 24 h before being embedded in paraffin. Paraffin-embedded thymus and spleen tissues were sectioned to 5-μm-thick sections and stained with hematoxylin and eosin (H&E). The stained tissue sections were examined under a microscope to observe any histomorphology changes (CX-21, Olympus Corporation, Tokyo, Japan).

The following are the steps taken to carry out the immunohistochemical detection of T lymphocyte subsets: paraffin sections were incubated with 3% H_2_O_2_ to deactivate endogenous enzymes. Then, sections were exposed to an anti-CD3^+^ antibody incubation (dilution 1:500, 17617-1-AP, Proteintech Group, Wuhan, China), anti-CD8^+^ antibody (dilution 1:500, 29896-1-AP, Proteintech Group, Wuhan, China), and anti-CD4^+^ antibody (dilution 1:1000, ab183685, Abcam United Kingdom) for two hours at room temperature. The secondary antibodies (diluted at 1:1000, ab6721, Abcam, UK) were added and incubated for 30 min at room temperature according to the manufacturer’s instructions. After dropwise staining with a newly configured DAB kit (A0050, Bio sharp, Hefei, China) and counterstaining with hematoxylin, positive regions of the sections were counted and analyzed using Image J-win64 software.

### 4.8. Microbiota 16S rRNA Pyrosequencing

Using the E.Z.N.A.^®^ Soil DNA Kit (Omega Bio-Tek, Norcross, GA, USA), total microbial genomic DNA was isolated from fecal samples and kept at −20 °C. An ABI GeneAmp^®^ 9700 PCR thermocycler (Applied Biosystems, CA, USA) was used to amplify the 16S rRNA gene V3 and V4 regions from DNA using the primers 338F (5′-ACTCCTACGGGAGGCAGCAG-3′) and 806R (5′-GGACTACHVGGGTWTCTAAT-3′). The purified amplification products were sequenced on an Illumina MiSeq PE300/NovaSeq PE250 platform (Illumina, San Diego, CA, USA) using the standard procedure of Shanghai Mayo Bio-pharm Technology Co., Ltd. (Shanghai, China). Using the UPARS software, sequences were clustered by OTU based on 97% similarity. The principal coordinate analysis (PCoA) plots, beta diversity, Venn map, hierarchical clustering, and alpha diversity calculations were performed using the gene cloud data analysis platform (https://www.majorbio.com, accessed on 12 January 2023).

### 4.9. Data Analysis

The mean and standard deviation (SD) of measurements made in triplicate were used to represent all values. The SPSS statistical program (version 27.0) was used to examine all statistical calculations. Statistical analysis was performed using one-way ANOVA followed by the Tukey test to determine group differences. Differences among the groups were analyzed by one-way ANOVA with the Tukey test. *p* < 0.05 was considered statistically significant, and *p* < 0.01 was considered highly statistically significant.

## 5. Conclusions

The current study found that RPPs significantly increased immune organ indices against HC-induced immunosuppressed mice. Meanwhile, RPPs could counteract immunosuppression by increasing the leukocyte counts, the expression of IL-2, IL-6, TNF-α, and INF-γ, as well as the expression of T lymphocyte subsets. Furthermore, RPPs could restore the morphology of the spleen and thymus. Moreover, RPPs indirectly regulate the immune function by regulating the balance of intestinal microbes, which increases the abundance of beneficial bacteria and decreases the abundance of harmful bacteria. Our research gives RPPs a theoretical foundation for usage as immunomodulators in functional foods.

## Data Availability

All data that support the findings of this available from the corresponding author on reasonable request.
